# Domestic Violence against LGBTI People: Perspectives of Portuguese Education Professionals

**DOI:** 10.3390/ijerph20136196

**Published:** 2023-06-21

**Authors:** Edgar Sousa, Sofia Neves, Mafalda Ferreira, Joana Topa, Cristina Pereira Vieira, Janete Borges, Rodrigo Costa, André Lira

**Affiliations:** 1 Plano i Association, ÍRIS Project, 4050-086 Oporto, Portugal; edgarsousap@icloud.com (E.S.); maf_gonf@hotmail.com (M.F.); jtopa@umaia.pt (J.T.); rodrigo.psy.uac@gmail.com (R.C.); andrebalio1@gmail.com (A.L.); 2 Department of Social and Behavioral Sciences, University of Maia, 4475-690 Oporto, Portugal; jborges@umaia.pt; 3 Interdisciplinary Centre for Gender Studies, University of Lisbon, 1300-663 Lisbon, Portugal; cvieira@uab.pt; 4 Public Health and Forensic Sciences, Medical Education Department, Faculty of Medicine, University of Oporto, 4200-450 Oporto, Portugal; 5 Social Sciences and Management Department, Open University, 1269-001 Lisbon, Portugal

**Keywords:** LGBTI, domestic violence, discrimination, educational sector, training, qualitative research

## Abstract

Lesbian, gay, bisexual, trans and intersex (LGBTI) people are more likely to be exposed to domestic violence than the rest of the population. Using a descriptive qualitative methodology, 28 professionals from the educational sector, aged between 28 and 64 years old (*M* = 44.5), were interviewed to describe and understand their perspectives on the sector’s ability to support, intervene and get involved with LGBTI people and, particularly, with victims or former victims of domestic violence. Through a thematic content analysis, three main themes emerged: (i) life trajectories of LGBTI people; (ii) domestic violence perpetrated against LGBTI people; and (iii) training of the educational sector to intervene with LGBTI people. The results show that Portuguese education professionals are not trained to recognize and intervene with LGBTI people and, in particular, with those who are victims of domestic violence, since they are unaware of the existence of protocols and/or guidelines for detecting and preventing risk situations among students. Furthermore, the curricular plan fails in the coverage of domestic violence and LGBTI-related topics, although the National Strategy for Citizenship Education has been implemented in Portugal since 2017. Findings suggest the need to invest in education professionals’ training.

## 1. Introduction

For forty-eight years, between 1926 and 1974, Portugal lived under *Estado Novo*, an oppressive dictatorship marked by the lack of personal, social and political freedom [1]. This explains why the decriminalization of homosexuality occurred only in 1982 and why the LGBTI movement and associativism only gained expression in the second half of the 1990s [2,3,4,5].

Throughout the first two decades of this millennium, Portugal has made significant progress in terms of recognizing the rights of LGBTI people [6,7,8], including the legalization of domestic partnerships between same-sex couples (2001), same-sex marriage (2010), protection of gender identity (2011), co-adoption by same-sex couples (2016), self-determination of gender identity and expression of trans people over 16 years old, legal protection of sexual characteristics at birth (2018) and the prohibition of discrimination in eligibility to donate blood (2021) [9,10,11,12,13,14]. 

Integrated into the National Strategy for Equality and Non-Discrimination 2018–2030 (ENIND) and aligned with the 2011 Istanbul Convention [15], Portugal has set out for the first time an Action Plan to Combat Discrimination based on Sexual Orientation, Gender Identity and Expression and Sexual Characteristics. This plan aims to grant visibility to LGBTI people by enlightening and educating society about their lives and real needs, as well as prevent and combat all forms of discrimination and violence against LGBTI people [16].

Despite these historical, social and legal developments [17,18], LGBTI people continue to be victims of discrimination and violence, both in the public and private spheres. At social, family, work, educational and legal levels, among others, asymmetries persist, which serves as a reminder that formal and legal rights do not always mean effective equality [19,20,21,22,23,24,25].

One of the most pervasive forms of violence against LGBTI people is domestic violence [23,26]. According to article 152 of the [Portuguese] Penal Code, domestic violence is defined as any act that occurs when an individual “repeatedly or not, inflicts physical or psychological abuse, including corporal punishment, deprivation of liberty, sexual crimes or prevents access or enjoyment of their own or common economic and patrimonial assets” [27]. In 2019, the [Portuguese] National Observatory of Discrimination against LGBTI+ People received 171 complaints, with 135 of them identifying the relationship between the victims and the aggressors. In 16.29% of the cases, victims disclosed that they have or had a close relationship with the aggressor or aggressors, whether as a family members, partners or former partners. Psychological and/or emotional violence (80%), physical violence (50%), deprivation of communication with other people (50%) and deprivation of liberty (40%) are the most reported typologies of domestic violence [21]. 

LGBTI people are frequently exposed to family rejection during their adolescence, when they go through a *coming out* process [28]. This rejection can take the form of social isolation, when their family uses manipulative or forced tactics to isolate them from the world [29]. *Outing*—the act or threat of publicly disclosing that a person is LGBTI without that person’s consent—is another LGBTI-targeted manipulative aggression mentioned in the literature, as it reinforces the power dynamic between the aggressor and the victim and keeps the latter in the relationship, due to fear that other people (e.g., family, friends, co-workers) will discover the fact that they are LGBTI [29]. According to Pereira [18], family support is important for LGBTI people to build good self-esteem and avoid depressive symptoms, especially for trans people. However, in general, LGBTI people grow up in a context of insult, isolation, invisibility, harassment, abuse and/or violence, thus summing their vulnerabilities [30,31,32]. Neves [33] argues that since there are specific forms of violence perpetrated against LGBTI people, the intervention must also be specific and offer an adequate response to these needs. Aware of this reality, the Portuguese government has been investing in the creation of specialized care and support structures and services for LGBTI people who are victims or former victims of domestic violence. Since 2016, these services are provided and promoted by ILGA Portugal and Casa Qui, both in Lisbon, and Plano i Association, in Matosinhos, which are all non-governmental organizations qualified to intervene with LGBTI people.

In this regard, professionals of the education sector also have a fundamental role in detecting and preventing situations of violence, whether at the family level or in intimate relationships [34,35]. Educational institutions are one of the main agents of socialization for children and young people [35,36], having a fundamental role in their development, [36,37,38]. At the same time, however, these institutions must provide “a solid education in values that includes learning about peace and non-violence” [39] (p. 59). Given that children and young people spend most of their day in schools [37], it is crucial to understand the vision and knowledge the professionals in this sector have about this social phenomenon [40]. Daily knowledge and practices can help perceive and identify cases of domestic violence perpetrated against LGBTI people as well as prevent them from happening.

The aim of this study is to describe and understand the perception and knowledge of professionals from the Portuguese education sector about the sector’s ability to support, intervene and get involved with LGBTI people, particularly in the case of victims or former victims of domestic violence. 

## 2. Materials and Methods

### 2.1. Study Design

This study employs a descriptive qualitative methodology, since it allows the description and understanding of complex social phenomena based on the experience of the protagonists [41,42]. This paper was carried out according to the Consolidated Criteria for Reporting Qualitative Research (COREQ) [43], allowing for greater transparency, rigor and methodological reproducibility.

### 2.2. Participants

This study took place in Portugal between February 2020 and April 2021. The study was disclosed among several educational entities, including elementary schools, high schools, universities and specialized professionals, and it was recognized by the Commission for Citizenship and Gender Equality in Portugal. The participants were selected using an intentional sampling technique, meeting the following selection criteria: (i) working or have worked in the education sector; (ii) intervening or have intervened directly or indirectly with LGBTI people; and (iii) intervening or have intervened directly or indirectly with LGBTI victims or former victims of domestic violence. The exclusion criteria were: (i) not fulfilling the inclusion criteria; and (ii) not providing an informed signed consent. Their sociodemographic characteristics are described in Table 1.

### 2.3. Data Collection and Procedures

Due to the COVID-19 pandemic, data collection was carried out online. The individual, in-depth and semi-structured interviews were conducted using the virtual video conferencing platforms *Google Meet* and *Zoom*. Each of the 28 professionals from the education sector was interviewed once only, for 56 min on average. The interviewers were part of the scientific research team and followed a semi-structured script with relevant questions (see Table 2). The choice of the interviewers was made according to their experience with LGBTI victims of domestic violence and for being trained in domestic violence and prevention of victimization or re-victimization. Before commencing the interviews, the interview protocol was explained, the informed consent was signed and the sociodemographic data were collected. These interviews were recorded in Portuguese, in audio format and were transcribed verbatim. These were later translated into English, for publication purposes. In the end, the participants had the opportunity to read the transcripts so they could verify the content.

### 2.4. Ethical Considerations

During the entire duration of this study, all ethical norms were respected to guarantee anonymity and confidentiality and to safeguard the rights and well-being of all the participants who authorized their participation by signing informed consent. This encompasses the rights and duties of the participants as well as the researchers. As a means of guaranteeing the protection of the data, codes were assigned to the interviews carried out.

The study was approved and co-funded by the Operational Program for Social Inclusion and Employment and has followed the Code of Ethics and Deontology of the Order of Portuguese Psychologists, the ethical principles of the American Psychology Association and the General Regulation on Data Protection from the European Union.

### 2.5. Data Analysis

Data collected were duly transcribed with the support of the *NVivo Transcription* software and reviewed a posteriori by the entire scientific research team [44]. The collected data were analyzed through the several phases of the Thematic Content Analysis method proposed by Braun & Clarke [45]: (i) Familiarization with the data: the complete reading of the transcripts and noting of the main ideas; (ii) Systematic generation of initial codes; (iii) Themes formation: assigning codes and grouping them according to the main ideas; (iv) Theme review: verifying the coherence between themes and their respective codes; (v) Definition and domination of topics; and (vi) Elaboration of the results: producing the final report with a selection of the themes and sub-themes related to the goal of this study.

## 3. Results

Through an inductive analysis, our study findings identified three main themes and eight sub-themes, which will help to understand the perception and knowledge of professionals in the education sector about the phenomenon of domestic violence perpetrated against LGBTI people.

### 3.1. Life Trajectories of LGBTI People

The first theme of this study focused on the description and understanding of the life trajectories of LGBTI people. Participants were asked to share their understanding and perceptions of what it could mean to grow up as an LGBTI person in Portugal. They were also asked to refer and describe key moments and struggles these trajectories may present.

#### 3.1.1. Plural Life Trajectories of LGBTI People

According to the interviewees, the life trajectories of LGBTI people are described as plural and diverse. The concept of plural life trajectories refers to the multiple groups of belonging and identities LGBTI people may fit in, rather than their individual unique experiences, and how these shape their experiences. This multiplicity encompasses the different sexual orientations, gender identities, gender expressions and/or sexual characteristics, as well as their intersectionality with other internal and external factors (e.g., age, ethnicity, functional diversity, geographic area where they live).

“In fact, there is such a great diversity of trajectories [that] […] it is not possible to identify a pattern of trajectories of lesbian women or bisexual women, in the same way, that it is not possible to identify a pattern of gay men or bisexual men. Neither will it be possible [to identify a pattern], nor will the experiences of trans people be similar, in particular, [the experiences] of young trans people today from [the experiences] of people who came out thirty years ago”.

(C., age 46, teacher and researcher)

Of all the LGBTI people, the participants highlighted the life trajectories of trans people and intersex people as the most discriminated against, violated and made invisible, warning about a total ignorance of the educational sector and of society itself, of the issues related to intersex people.

“There is a particular trans person whom I have been following for some time, and it bothers me because there is not a session—and we have sessions every fortnight—there is not a single session where she does not bring up an experience when she was significantly discriminated”.

(D., age 29, clinical psychologist and researcher)

“I think that, right now, […] trans issues are the most complicated, because I still think we are in the pre-history of intersex. Because they are not seen, [and] they are not talked about. And, certainly, the statistics tell us they exist, but I have not met any […]. That is an indicator that things are even more silenced”.

(M., age 58, teacher)

#### 3.1.2. Possible Transversal Aspects to the Life Trajectories of LGBTI People

Despite the opinions that the life trajectories of LGBTI people are marked by a plurality of experiences, the participants recognize the existence of some experiences that may or may not be transversal to most of the life trajectories of people LGBTI, which can occur in different forms, dimensions and degrees. One of these experiences is internalized LGBTIphobia. This phenomenon is present in the life trajectories of several LGBTI people who, during their growth, were exposed to various stereotypes and prejudices of society for being LGBTI ended up internalizing some of them and creating hatred or rejection towards their own sexual identity, which ultimately nullifies their self-acceptance.

“What happens for many of the people with non-heterosexual sexual orientations or non-cisgender gender identities […] is that they often nullify their [own] identities, nullify their self-acceptance so that in some way [their identity] is accepted by other people. And that is where the danger lies and what interferes in their development, their self-construction, their self-care [and] their self-acceptance”.

(L., age 36, researcher)

“The overwhelming majority of us, kids, do not know what it is like to be gay, what it is like to be lesbian, what it is like to be bisexual, what it is like to be a trans person or what a heterosexual person is. We do not know that, but from a very early age, we learn that “dyke” and “faggot” are bad things and, therefore, [that is] the first contact we have with this identity, which may or may not be ours. It is an identity that for a long time was prohibited, for a long time was considered a mental disorder, was persecuted, was criminalized. Homosexuality was criminalized in Portugal until 1982—and that is why this is the first contact that children have, regardless of sexual orientation, gender identity or gender expression”.

(S., age 44, researcher)

Another aspect mentioned by the participants is the rejection and violence by family members and/or society, which can lead LGBTI people to having fewer support networks and to feel that their sexual identity is minimized, seen as a “phase” or not as valid as that of heterosexual and cisgender people.

“In a first impact, it is rare for families to react well. […] For example, the parents calmly accepted the fact that this young man, whom they see as a girl, […] is attracted to girls, therefore a lesbian in their minds. They accepted it perfectly, yet, in reality, he is a straight trans person, and this part they did not [accept]. It is a curious thing […] Trans issues are not very easy. The issue is more complicated. People make up a lot of stories because they think it might still be a phase”.

(J., age 31, psychologist)

“[…] these people are not necessarily born—they might be—but they are not necessarily born in families with these experiences. And, therefore, the most direct microsystem of socialization and promotion of development is a microsystem that does not support. [It] does not support in the sense that […] none of the people in that family are trans, none are gay or lesbian, and, therefore, [a microsystem] in which there is this additional challenge for the child. [They] have to go through this process of involvement and socialization as someone who is singular in this family, even if the family is very supportive”.

(C., age 46, teacher and researcher)

“We always count for less. I talk about this a lot with my friends who are, or have been, in relationships with people of the same sex. And, in fact, when their relationships end, among friends or family, it always matters a little less. If they were a [straight] man or a woman who just got divorced, it would have a much greater impact, and, as a couple of people of the same sex, either two men or two women, that relationship is always diminished somehow. As if our suffering and our relationships are actually lesser, when in reality it is a bit the other way around”.

(S., age 44, researcher)

According to the analysis of the speeches, the life trajectories of LGBTI people are described by the participants as difficult, given that there is a strong presence of situations of insult, isolation and invisibility in their lives. These situations help reinforce the mechanisms of discrimination, stigmatization and violence against LGBTI people in society.

“The general thing, clearly, is the experience of insult, […] an insult that does not have to be direct. […] Growing up with insult, growing within the insult. So […] what happens in terms of identity is creating an identity based on insult and understanding the insult before understanding the identity. This is general among LGBT people—and I say this to everyone—and this is a very difficult experience, difficult indeed. It is much more difficult […] the greater the invisibility around them, the lesser possibility of establishing mirrors, of finding mirrors. But also, the issue of isolation itself, that is, whether or not there are people with whom to share experiences of identity construction and experiences of discrimination. […] This insult exists before we exist as people”.

(P., age 46, teacher)

The participants also mentioned that LGBTI people are often exposed to experiences of discrimination by professionals from different sectors and services, such as professionals in the health and education sectors.

“I think one of the most prevalent things is related to having prior experiences with professionals that were not very positive in terms of prejudice and discrimination. Well, my experiences are actually subtler things, not such direct forms of discrimination”.

(D., age 29, clinical psychologist and researcher)

“If the doctor himself, as much as the teacher, do not adjust their language to make it inclusive…. Because if a doctor asks me about my marital status and I tell her that I am single or if I tell her that I’m married and she assumes that I’m married to a woman, [she] should not assume that. Just as much as I should not assume in appointments with my patients, when it is the first time and I am collecting information, that they are heterosexual”.

(L., age 48, teacher and clinical psychologist)

The life trajectories of LGBTI people are accompanied by false expectations and/or difficulties associated with *coming out*. From the speeches of the participants, the false belief that everything is fine is verified, from the moment they reveal themselves to be LGBTI, they become more vulnerable to more active experiences of discrimination.

“Unrealistic expectations that «I come out [as LGBTI] and everything is fine» because then, what happens is that those who have not yet come out have fewer experiences of a more active discrimination”.

(D., age 29, clinical psychologist and researcher)

“She came out as a lesbian in her school context and at home. At home, it went very badly. Her parents used physical force to try and “normalize” her. At school she was always really well accepted, but there was a teacher who repeatedly made speeches such as «you can be whatever you want outside, but here you are X and act like a straight girl»”.

(J., age 38, teacher and researcher)

A final aspect highlighted in the interviews is that the participants affirm that LGBTI people need to establish contact with people with the same identity. This happens because, being people with similar experiences, they end up looking for other LGBTI people who can understand and support them in overcoming their needs and struggles.

“Especially among trans people, also due to their invisibility, there is a quite curious network of support between them. […] When people realize that it is important to meet and get to know more people—because what happens often is «It seems like I am the only one in the world»—and when they come to one or two meetings [in the support groups], trans people approach me and tell me «Well, I am already in the Facebook group. I am already in the WhatsApp group and that is all of Portugal». And I feel like […] they create a lot more networks between them. […] «Well, I understand it as well. This site is better to buy this binder. Look, do not do this! Be careful not to make it too tight. Look, when I went there the nurses were cool. Look, that place is terrible». There is this constant sharing of experiences. I find it positive and even helpful in solving doubts”.

(D., 29 years old, clinical psychologist and researcher)

### 3.2. Domestic Violence Perpetrated against LGBTI People

The second theme of this study focused on the description and understanding of the phenomenon of domestic violence perpetrated against LGBTI people. Participants were asked to share their understanding and perceptions on the phenomenon of domestic violence and how it affects LGBTI people. In addition, they were asked more specifically to share their perspectives on who the aggressors are, what the main needs are that LGBTI victims usually have and whether they considered it relevant to have specialized structures for the reception and/or intervention with LGBTI people victims of domestic violence.

#### 3.2.1. Domestic Violence Perpetrated by Family Members

Firstly, participants highlight specificities in the typologies and forms of domestic violence perpetrated against LGBTI victims by relatives. Some more direct examples of this violence are denial, silencing, invisibility, isolation and home expulsion, mainly when it comes to LGBTI children and youth, while the most indirect are, for example, commenting on news about LGBTI issues, without directly addressing the victim.

“For example, men whom I have followed said that when they came out to their parents, there was no drama, in the sense that there was no discussion, no yelling, yet there was silence from then on. Thus, their affective life ceased to exist and, consequently, it is as if it does not exist, which is a form of violence”.

(L., age 48, teacher and clinical psychologist)

“[…] coming out [as LGBTI] is followed by exclusion from the family. They are kicked out of their house, suffer stigmatization and even ridicule, or are marginalized by some family elements […]. Consequently, [this] leads to [LGBTI] people having, on average, a much smaller family and support networks, further making their lives harder in a number of ways, and [their] childhoods, in particular, marked by contexts of greater violence”.

(S., age 48, teacher and researcher)

“There are all kinds of violence, basically, physical violence, […] also examples of children, teenagers, who are locked in their rooms, unable to communicate with anyone”.

(P., age 46, teacher)

In addition to these examples of domestic violence, the participants shared episodes of discrimination and extreme violence of which they were aware, in which aspects such as the use of weapons against LGBTI children and youth or their submission to “conversion therapies” stand out.

[After mentioning LGBTI associations which they have worked for:] “I remember a girl whose father […] grabbed her and threw her out of the window onto the garden, and the glass broke. I remember this case specifically. I remember a father who pointed a shotgun at his son. I remember another situation of a father who pointed a knife at his son. I remember parents kicking their sons and daughters out of the house, but that was a long time ago. […] I imagine many of these situations still happen”.

(S., age 44, researcher)

“I remember this young man […] that when he talked to his parents about his sexual orientation […]—and I remember what he told me perfectly—he said, “my parents forced me to go to doctors, witches, and psychiatrists because they thought I needed to be cured”. For those parents, it was important to ‘cure’ their son, so he underwent a period of going to a multitude of doctors, witches and psychiatrists. One day, tired of that oppression and violence, he decides to take a friend home and tell them she was his girlfriend, solving the issue once and for all”.

(L., age 48, teacher and clinical psychologist)

#### 3.2.2. Domestic Violence Perpetrated in Current or Former Intimate Relationships

Regarding domestic violence in current or former intimate relationships, some specificities stand out. From the analysis of the speeches, the influence of aspects previously described by the participants, which mark the life trajectories of LGBTI people, such as invisibility, is understood. This will allow the aggressor to use *outing* as a threat strategy against the victim, in which they can not only threaten to reveal some aspect of the victim’s sexual identity but also other conditions, such as having HIV or AIDS, thus hindering the process of the revelation of the person as a victim, their request for help and the formalization of the complaint process.

“[…] the specificities of domestic violence in LGBT couples are even more drastic than in heterosexual couples, mainly because [as long as] the relationship is invisible or unknown to the people/community around the couple, I believe that threats may be focused on «I will tell everyone that you are gay, lesbian or bisexual»”.

(R., age 32, science management)

“[…] in a study on the prevalence of HIV/AIDS among men who have sex with other men, we found this accumulation of exclusionary and vulnerability vectors. […] And, for example, we found a lot of fear of revealing […] not only their sexual orientation but also the abuse one suffers in an intimate relationship. […] And often the offender will blackmail the victim with this”.

(I., age 55, teacher)

“With LGBT people, what happens is that, because they are already in place of stigma in relation to that group, often [LGBT] people tend to isolate themselves even more, just as it happens in heterosexual relationships, but they [also] tend to isolate their partner. However, when the person themself no longer has support networks, it makes the process of disclosure and support much more difficult. […] So, all of this makes the process more difficult in some ways, such as following up with the victims, or getting them to come forward”.

(L., age 36, researcher)

#### 3.2.3. Need for Specialized Care and Shelter Structures for Victims of Domestic Violence

The participants highlighted the need for specialized care and shelter structures for LGBTI victims of domestic violence. As they pointed out, LGBTI people continue to be victims of differential treatment, discrimination and violence and, as a such, society must promote care, shelter and support services that do not reinforce these discrimination processes, but rather mitigate and eliminate them.

“[…] in the current social and cultural context, the first step will be to create specific response services, especially among peers or people seen as peers, or people who have specific training. Above all, […] creating a physical space where people can go and where there is a community is key for these individuals… Unfortunately, in situations of violence, this is essential”.

(E., age 28, researcher)

“[…] When speaking about the present moment, I think the ideal scenario would be for all contexts to be able to serve anyone, regardless of any identity-related issue people may have. However, I believe that not everyone is capable of doing so. In fact, we have public cases of psychologists having completely anti-productive attitudes and behaviors regarding these issues. Therefore, having specialized services for people with these problems will give them more security […] knowing that they will find a competent professional […] to tackle the problem they are going through or the needs they have”.

(J., age 38, teacher and researcher)

“Universal and specific services can coexist, just as they do in any other area, right? There are emergency services, primary health care services and speciality services, after all. […] One does not replace the other. […] In a first phase, say, [services] to raise awareness of the specific needs of the LGBTQI population and develop competence and sensitivity for high-quality healthcare that carefully meets their needs. This first phase comprises the creation of specific services. These are not expected to take on all the care of the population. They are effectively specialized for specific issues and will coexist, at this stage, with universal care services. This does not mean that, in universal services, the professionals working there can be absolutely insensitive or have no training or competence in these areas”.

(C., age 46, teacher and researcher)

### 3.3. Training of the Education Sector to Intervene with LGBTI People

The third and last theme of this study focused on the description and understanding from the perspective of the participants, on the training of the educational sector to intervene with LGBTI people and, in particular, with those who are victims of domestic violence. The participants highlighted the lack of knowledge of protocols and/or guidelines in the education sector, as well as the poor training of professionals.

#### 3.3.1. Perception of Knowledge of Protocols and/or Guidelines in the Education Sector

During the interviews, the participants demonstrate that there is knowledge about specialized care and shelter structures for LGBTI victims of domestic violence in Portugal. However, when questioning the knowledge that other professionals have about national or international protocols and/or guidelines for working and/or intervening with LGBTI people and, in particular, with those who are victims of domestic violence, it seems that this knowledge is scarce or even non-existent in the educational sector.

“That knowledge, I do not think they have. […] When I say they do not have it, I am referring to the school community, that is, teachers, teaching assistants [etc.] […]. The fact that we are always working in collaboration with other associations […] helps us have enough knowledge to be able to provide support whenever [cases] arise”.

(V., age 28, sociocultural animator)

“Not in a consistent way, I think, […] even though it is institutionally required. I feel like changes happen by chance, depending on who is there. If it is someone more conscious of these issues, they will do what they can, perhaps proposing training, [or] presenting the guidelines”.

(D., age 29, clinical psychologist and researcher)

#### 3.3.2. Perception of Training in the Education Sector

For the participants in this study, training in their sector of activity to work and/or intervene with LGBTI people and, in particular, with those who are victims of domestic violence, is scarce. Most of the people interviewed pointed out that the educational sector is poorly prepared to work directly with LGBTI people in general, this being justified by the lack of training, time and inadequacy of the curricular plan for its lack of coverage of domestic and LGBTI-related topics, since these are not addressed in the classroom context.

“[…] I think that things have come a long way seeing that some teachers are already being trained in this area. However, it is still a rather problematic area, mainly because there are teachers with no training at all who continue following a very traditional, stereotyped and often religious paradigm. This occasionally interferes with their teaching practice and the way they address these LGBTI young people and handle conflicts”.

(J., age 38, teacher and researcher)

“There was a trans boy here at the school, […] and people made a fuss about him and his transition because it was something new, and these teachers had never heard of such a thing”.

(J., age 38, teacher and researcher)

“In education, […], there are so many problems and so many things to work on that teachers are up to their ears in work […]. That is their real problem rather than not being receptive”.

(M., age 58, teacher)

“I have no opportunity to integrate these topics into my students’ education, […] as the curriculum does not include them. I can only manage to introduce these topics very occasionally”.

(M., age 61, teacher)

In addition, they highlighted the lack of technical staff in the education sector, support offices in educational institutions for LGBTI people and the articulation of educational institutions with LGBTI associations.

“I believe it is poorly [prepared]. […] I think we lack resources and coordination between academia and universities. […] Schools [also lack] support offices for victims or LGBT populations, something that most of the time does not happen, and when it does, it is very sporadically”.

(S., age 48, teacher and researcher)

“In a school or a school cluster, the number of technicians is so reduced—unfortunately—that we end up intervening from a standpoint of remediation. Meaning that we have to respond to needs. It is an immediate response. […] At school, we have the freedom to approach these topics, but we tend to focus on the ones that arise”.

(V., age 28, sociocultural animator)

## 4. Discussion

This study aimed to describe and understand the perception and knowledge of professionals from the Portuguese education sector about the sector’s ability to support, intervene and get involved with LGBTI people, particularly with victims or former victims of domestic violence. Given the fact that family and educational institutions are often unsafe environments for LGBTI people [20,46], with children and youth being exposed to severe situations of discrimination and violence, it is imperative to explore how to improve professionals’ skills and capacitate schools to detect, prevent and combat it, considering their privileged position of close contact and influence in the development of LGBTI people [37].

According to the results found in the present study, the realities of LGBTI people become invisible both in the educational and domestic context. The 2022 Preliminary Report on LGBTQ+ Youth and School Environment in Portugal, which comprised a sample of 1535 students, elucidates the dimension of the issue. Out of the students who identified as LGBTQ+, only 50.5% had someone in their family who knew their sexual orientation, whilst 38.1% had no one. In regard to gender identity, only 13% felt accepted by everyone in the family whereas, almost at the same proportion, 9.7% felt no one in their family accepted them. As for coming out in the school context, only 13% of the LGBTQ+ students have come out to their entire class, and merely 1.8% have done so to professionals in the education sector. One final aspect of this preliminary report to be highlighted is the lack of representation in the school context, in which only 13% of the students knew someone or some professional in the education sector who openly identified as LGBTQ+, and 40.6% reported that issues associated with LGB people were never addressed. This figure is even higher (54.2%) when referring to issues related to trans people [46].

Following the evidence of other national and international studies (e.g., [5,20,32]), this study concludes that the realities of LGBTI people are frequently invisible both in the domestic and educational context, bringing attention to the trajectories of trans and intersex people who tend to suffer increased vulnerability to discrimination, violence and invisibility in society. In fact, despite being subjected to different types of discrimination and violence during their lives, LGBTI people’s belonging and identities make particular their experiences of oppression. The present study traces some aspects that may or may not be transversal to the majority of LGBTI people. These may occur in different forms, dimensions and degrees, namely: (i) internalized LGBTIphobia; (ii) rejection and violence by family members; (iii) experiences of insult, isolation and invisibility; (iv) experiences of discrimination; (v) false expectations and/or difficulties associated with *coming out*; and (vi) the need to be in contact with people with the same identity.

According to participants, family members, partners and former partners are a significant source of violence to LGBTI people. Concrete examples of the violence perpetrated by relatives include: (i) expelling LGBTI people from their homes; (ii) isolating them; and/or (iii) submitting them to “conversion therapies”. Concerning this last example of violence, the Order of Portuguese Psychologists [47] and the College of Psychiatry Specialty of the Order of Physicians [48] disapprove of this type of intervention with LGBTI people, stating that it can contribute to an even more negative impact on their lives, as in terms of mental health. When it comes to violence within current or former intimate relationships, aggressors use various strategies to control and intimidate their victims. Some of the mentioned strategies are isolating the victim, thus weakening their social support network, or *outing*, a threat to publicly reveal a victim’s sexual identity characteristic [5,29].

Despite Portuguese education professionals being aware of the difficulties LGBTI people face since childhood, in multiple domains, they lack training, knowledge and resources to effectively intervene. Not only are they poorly prepared to work or provide intervention for LGBTI people [46], but they are also unaware of the existence of directives, protocols and/or guidelines within the education sector, to work and/or intervene with LGBTI people and, particularly, with LGBTI victims of domestic violence, and guidelines and best practice guides developed by national associations and state bodies (e.g., [29,47,49,50,51,52]). The problem lies not in creating new resources but, most likely in distributing and diffusing the ones already in place.

Participants highlighted the inadequacy of the curricular plan for its lack of coverage of domestic violence and LGBTI-related topics, lack of time to address those issues and the limited number of technical staff in the education sector. These questions are particularly relevant as the National Strategy for Citizenship Education and the Action Plan to Combat Discrimination based on Sexual Orientation, Gender Identity and Expression and Sexual Characteristics has been implemented in Portugal since 2017 and 2018, respectively [16,53]. The evaluation of the efficacy of these mechanisms must be addressed to eventually adjust and enhance their execution.

Moreover, the study has revealed that professionals in the education sector are poorly prepared to work or provide intervention for LGBTI people [46], and in particular, for those who are victims of domestic violence. Similarly, the 2019 LGBTI Education Project Report concluded that only 48.1% of professionals in the education sector feel capable of speaking about LGBTI issues, 59.3% felt moderately knowledgeable about the resources made available in educational institutions to address LGTBIphobia and 66.7% demonstrate the need for specific training regarding LGBTI issues [54]. Additionally, the European project, Diversity and Childhood, concluded that 55.6% of professionals in the education sector do not have access to resources in the institutions where they work and that 73% do not have specific training that allows them to support LGBTI people [55].

Otherwise, and contrary to other documents (e.g., [50]), the results of this study have indicated that the participants reveal some knowledge about the life trajectories of LGBTI people. Yet, taking into account the disqualification of the education sector itself and the fact that LGBTI people are made invisible within it—sometimes victims of their own invisibility, with four out of five hiding their sexual orientation and/or gender identity from professionals in the institutions they attend [46]—this study demonstrates that these professionals lack specialized training in order to identify, intervene and/or prevent situations of domestic violence against LGBTI people.

## 5. Conclusions

Despite the historical evolution and important advances in terms of recognizing the rights of LGBTI people in Portugal, these have not yet been effectively translated into their lives. Even now, LGBTI people continue to be abused and discriminated against based on their sexual orientation, gender identity, gender expression and/or sexual characteristics in both family and school contexts, which should be safe, inclusive and supportive environments for LGBTI children, young people and adults. Therefore, there is the necessity to actively intervene in the Portuguese education sector and provide teaching and non-teaching staff with knowledge, tools and resources on LGBTI issues, especially trans and intersex issues, given how invisible they are in society. The reason is that LGBTI people at risk seek help and turn to people they perceive as safe. Professionals in the education sector are often seen as these trustworthy figures due to the extended and close contact they have with students.

One of the main conclusions is that academia and civil society should be advised to increase research on trans and intersex people, both in terms of their life trajectories and the phenomenon of domestic violence perpetrated against them in family contexts and intimate relationships. Moreover, as guidelines for future studies, it is suggested that studies aim for a deeper understanding of the reality and the impacts produced by the COVID-19 pandemic on LGBTI people, and, in particular, those victims of domestic violence. Although the present study obtained contradictory information that LGBTI people reported less, other studies indicate the opposite. Moreover, since much of the previous research on LGBTI people and the education sector has focused mainly on LGBTIphobic bullying, it is also proposed that future studies explore the role of professionals in the education sector in situations of domestic violence against LGBTI people and its prevention.

Regardless of all the investment in research, to eradicate discrimination and violence perpetrated against LGBTI people, and in particular, against those victims of domestic violence, it is indispensable to promote social change and, consequently, a shift in the paradigm of education that must involve the training of professionals in the Portuguese education sector, as well as familiarizing them with the national and international action protocols. Thus, the Portuguese education sector should facilitate and promote training actions among professionals who work directly or indirectly within this sector to not only foster the dissemination of inclusive practices that can be used daily to combat stereotypes and prejudices but also provide professionals with useful tools to identify, intervene and prevent situations of domestic violence perpetrated against LGBTI people.

## 6. Study Contributions and Limitations

This study presents itself as a pioneer in the Portuguese context, due to the fact that it specifically studies the life trajectories of LGBTI people who are victims of domestic violence, based on the perspectives of one of the main agents of socialization in society.

One of the limitations of this study is that it took place during the COVID-19 pandemic, which meant the interviews had to be conducted online. Despite the numerous advantages of virtual interviews, such as the possibility of connecting to participants from different regions of the country, this format also requires additional attention from the research team to ensure they capture the participants as spontaneously as possible in their responses since nonverbal language has a lesser impact.

Another limitation was to do with the fact that some participants believe they do not work directly with LGBTI people. As the previously mentioned studies confirmed, LGBTI people often are made or make themselves invisible in educational contexts. Therefore, there is a possibility that some participants gave biased answers based on the assumption that they do not work directly with LGBTI people when in fact they may do and not be aware of it.

## Figures and Tables

**Table 1:** Sociodemographic characteristics of the participants (*n* = 28).

Sociodemographic Characteristics	Frequency (%)
Age	
Range: 28–64 (*M* = 44.5; *SD* = 10.57)	
Gender	
Woman	19 (32.1%)
Men	9 (67.9%)
Nationality	
Portuguese	28 (100%)
Marital status	
Single	14 (50%)
Married	8 (28.6%)
Divorced	5 (17.9%)
Domestic partnership	1 (3.6%)
Education	
Social sciences and humanities	23 (82.1%)
Educational sciences	3 (10.7%)
Legal sciences	1 (3.6%)
Other areas	1 (3.6%)
Training on LGBTI issues	
Yes	15 (53.6%)
No	13 (46.4%)
Geographic area of work	
Lisbon metropolitan area	12 (42.9%)
North	11 (39.3%)
Center	5 (17.9%)
Work directly with LGBTI people	
Yes	18 (64.3%)
No	10 (35.7%)

**Table 2:** Interview script.

Stage	Questions
Introduction	Presentation of the study and research team to the participant were established, as well as acknowledgement of their participation. Doubts were clarified, informed consent was signed and participants were asked to introduce themselves in order to sociodemographic data could be collected.
Development	The interview took place after the sociodemographic data were collected. The following questions were asked with each of the people interviewed, and some others may have been added individually and uniquely to each interview, depending on the topics raised by each person: (i) How are life trajectories of LGBTI people characterized?; (ii) In a situation of domestic violence perpetrated against LGBTI people, what needs do the victims usually have? Do these needs vary according to gender identity and/or expression, sexual orientation or sexual characteristics?; (iii) Do you consider necessary the existence of specialized structures for the reception and/or intervention with LGBTI people who are victims of domestic violence?; (iv) Do you know protocols or guidelines for intervention with LGBTI people who are victims of domestic violence?; (v) Is the education sector trained to intervene with LGBTI people who are victims of domestic violence?
Conclusion	A last open question was asked, with the aim of collecting additional information that the participant might find relevant “Is there any other aspect that you would like to highlight?”. The interview ended with a reinforcement of acknowledgement of their participation.

## Data Availability

The participants of this study did not give written consent for their data to be shared publicly, so due to the sensitive nature of the research supporting data is not available.
